# Camelid VHHs Fused to Human Fc Fragments Provide Long Term Protection Against Botulinum Neurotoxin A in Mice

**DOI:** 10.3390/toxins11080464

**Published:** 2019-08-07

**Authors:** Svetlana A. Godakova, Anatoly N. Noskov, Irina D. Vinogradova, Galina A. Ugriumova, Andrey I. Solovyev, Ilias B. Esmagambetov, Amir I. Tukhvatulin, Denis Y. Logunov, Boris S. Naroditsky, Dmitry V. Shcheblyakov, Aleksandr L. Gintsburg

**Affiliations:** 1 Department of Genetics and Bacteria Molecular Biology, Gamaleya Research Center of Epidemiology and Microbiology, 18 Gamaleya Street, Moscow 123098, Russia; 2 Department of Bacteriology, Gamaleya Research Center of Epidemiology and Microbiology, 18 Gamaleya Street, Moscow 123098, Russia; 3 Department of Medical Microbiology, Gamaleya Research Center of Epidemiology and Microbiology, 18 Gamaleya Street, Moscow 123098, Russia

**Keywords:** camelid single-domain antibodies, VHH, *Clostridium botulinum*, toxin neutralization, phage display, dimers, Fc fragments

## Abstract

The bacterium *Clostridium botulinum* is the causative agent of botulism—a severe intoxication caused by botulinum neurotoxin (BoNT) and characterized by damage to the nervous system. In an effort to develop novel *C. botulinum* immunotherapeutics, camelid single-domain antibodies (sdAbs, VHHs, or nanobodies) could be used due to their unique structure and characteristics. In this study, VHHs were produced using phage display technology. A total of 15 different monoclonal VHHs were selected based on their comlementarity-determining region 3 (CDR3) sequences. Different toxin lethal dose (LD_50_) challenges with each selected phage clone were conducted in vivo to check their neutralizing potency. We demonstrated that modification of neutralizing VHHs with a human immunoglobulin G (IgG)1 Fc (fragment crystallizable) fragment (fusionbody, VHH-Fc) significantly increased the circulation time in the blood (up to 14 days). At the same time, VHH-Fc showed the protective activity 1000 times higher than monomeric form when challenged with 5 LD_50_. Moreover, VHH-Fcs remained protective even 14 days after antibody administration. These results indicate that this VHH-Fc could be used as an effective long term antitoxin protection against botulinum type A.

## 1. Introduction

Botulinum neurotoxin (BoNT) is the strongest organic poison for humans and animals. It is produced by the anaerobic, Gram-positive, spore-forming, rod-shaped bacterium *Clostridium botulinum* [1,2]. The estimated human lethal dose is about 10 nanograms per kilogram of bodyweight if the toxin is inhaled and one microgram if it is taken orally [3,4]. The most common forms of natural botulism are food-borne, wound, and infant [2]. Food-borne botulism occurs through contaminated food ingestion, and the case fatality rate is now about 5–10% in developed countries (as opposed to 60–70% before 1950). Wound botulism occurs when an open wound is exposed to *C. botulinum* spores, and the case fatality is approximately 10–15% of patients even with aggressive treatment. Infant intestinal botulism occurs with spores ingested and spread over the digestive tract, as infants lack the protective flora of adults, with case fatality rate estimated to be le,ss than 1–2%. Inhalation botulism does not occur naturally and may occur in the context of a bioterrorist attack [2,5]. Among the four major human pathogenic BoNTs (BoNT/A, B, E, and F), BoNT/A poses the most serious challenge for medical treatment due to its extremely high potency and extraordinary persistence in human patients [6]. According to the World Health Organization [7], an antitoxin, usually based on equine antitoxin, should be administered as soon as the diagnosis is made. However, side effects, such as allergic reactions, fever serum sickness, and anaphylactic shock, often occur. In the past, at-risk persons and populations have been vaccinated with a chemically inactivated penta-serotype BoNT/A-E toxoid [8]; however, its use has been discontinued due to declining potency [9]. In other affected individuals, botulism treatment is mainly supportive, with mechanical ventilation being the only effective life-saving treatment [10,11].

A good alternative to antitoxin serum with minimal or no side effects is treatment with monoclonal antibodies (mAbs) to BoNT, which can be prduced in vitro. Along with conventional antibodies, single-domain antibodies (sdAb), also referred to as VHHs (variable domains of heavy-chain only stibodies) or nanobodies, have been widely used since their discovery in camelids along with classical immunoglobulins (IgG) [12,13,14]. The absence of light chains in IgG and a lack of constant domain 1 (CH1) in the heavy chain are the key characteristics of heavy-chain antibodies (HC-Abs). Therefore, an antigen-binding site of HC-Abs is formed only by a single domain, which is linked directly via a hinge region to the Fc (fragment crystallizable) domain. HC-Abs recognize the antigen with only one special variable domain, referred to as VHH. The structure of the VHH domain resembles the VH IgG domain [15]. The complementarity-determining region 3 (CDR3) of these HC-Abs possesses the extraordinary capacity to form long finger-like extensions, which can extend into cavities on antigens, as the CDR3 is often much longer than that of conventional VH domains [16,17]. Despite their small size (~15 kDa), sdAbs maintain affinities and antigen-binding specificities comparable to those of full-size mAbs [18]. Clear advantages include the ability to recognize hidden antigenic sites that are inaccessible for conventional antibodies due to their structure; stability over a wide range of temperatures and pH; high solubility, as well as economical and facile expression and production in large quantities in microorganisms [15,17]. Several studies were conducted to test the potency of VHHs as inhibitors of viral infections [18] and different toxins, produced by such plants and microorganisms as *Ricinus communis* [19], *Mycoplasma hominis* [20], *Clostridium tetani* [21], *Clostridium difficile* [22], *Crotalus durissus terrificus* [23], and Shiga toxigenic *Escherichia coli* (STEC) [24]. It has been demonstrated that the antigen-binding region of VHHs produced by camelids showed strong anti-BoNT activities in animal models [25,26]. Due to their unique features and high efficacy, sdAbs are currently in clinical phase trials for the treatment of a wide range of diseases, including cancer, inflammation, hematology, and respiratory diseases [27]. For instance, Cablivi is a nanobody-based medicine, which has recently been approved by the Food and Drug Administration (FDA) for acquired thrombotic thrombocytopenic purpura (aTTP) treatment [28].

However, the advantages of VHHs may also become their impediments. Their small size enables good penetration into tissue and rapid distribution, but their short half-life in the blood circulation limits the time for interaction with hard-to-reach epitopes and for crossing endothelial barriers in sufficient amounts [27,29,30]. Nevertheless, due to their relatively simple structure, VHHs can be optimized by genetic engineering to obtain desired properties, including extended half-lives [29].

One approach was to add an albumin-binding peptide to the C-terminus of VHH anti-botulinum, which increases the serum half-life of the VHH to 1–2 days [31]. Another approach was to generate virus vectors encoding chimeric proteins with one or more VHHs fused in frame to a cDNA encoding the red blood cell membrane proteins glycophorin A or Kell. In vitro studies and stem cell transplantation research has demonstrated that the half-life of these VHHs could be extended to several weeks with retained functionality [32]. The VHH could also be fused to the fragment crystallizable (Fc) region of an IgG. Such modifications, termed fusionbodies, increase the total protein complex size, as well as VHH-Fc interaction with neonatal Fc-receptor (FcRn), making it impossible to clear by the renal filtration system [30]. Furthermore, the attached Fc fragment can promote effector functions, such as phagocytosis and cytotoxicity, thereby enhancing the neutralization of pathogen entry and replication [33].

In this study, we focused on screening specific BoNT/A neutralizing alpaca VHHs from a VHH antibody immune library by phage display using BoNT/A and BoNT/A treated with dithiothreitol (BoNT/A-DTT) as antigens. Two clones (B11 and G3) with high neutralizing potency at lethal dose (50 LD_50_) were obtained in phage forms. These clones were produced as VHHs, which were then modified as dimers or fused with human IgG Fc-fragments (VHH-Fc, fusionbody). The VHH-Fc modification greatly increased their neutralizing potency and serum half-life.

## 2. Results

### 2.1. Generation of VHHs to BoNT/A

**Figure 1:**
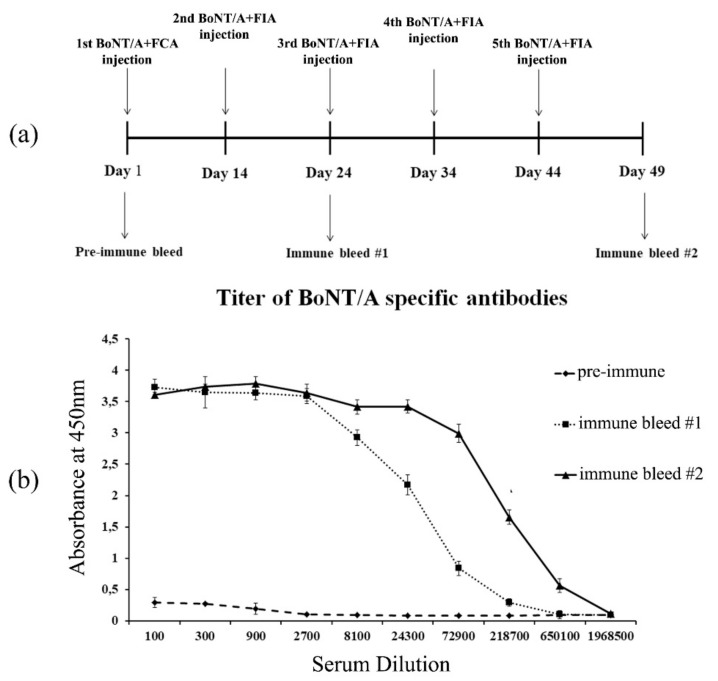
(**a**) Botulinum neurotoxin (BoNT)/A was used as an antigen with Freund’s complete adjuvant (FCA) and Freund’s incomplete adjuvant (FIA) for alpaca immunization following the schedule shown; (**b**) ELISA analysis showing the antibody signal from pre-immune and immune alpaca sera collected on days 24 and 49 post-immunization. Triplicate measurements were averaged.

Alpaca immunization was performed using five sequential injections with an interval of 14 days between the first and second immunizations and 10 days between all subsequent immunizations to generate an immune library of single-domain antibodies (Figure 1a). After 24 and 49 days post-immunization, blood was collected, and the BoNT/A specific antibodies titer was measured by enzyme-linked immunosorbent assay (ELISA). The immune serum showed a clear response to BoNT/A (Figure 1b). Before immunization, all sera showed the background levels of antibody titers. The final titer of toxin-specific antibodies in the alpaca serum was more than 1/600,000 as the result of the five-fold immunization scheme.

### 2.2. VHH Library Construction and In Vivo Polyclonal Neutralization Verification

**Figure 2:**
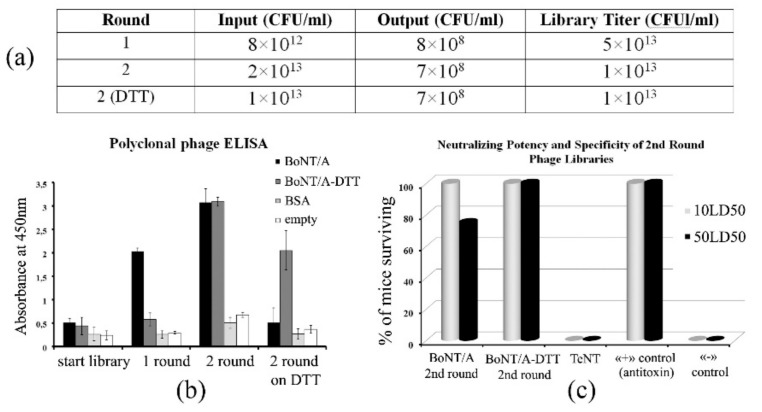
(**a**) Library biopanning summary, showing the round of selection, input and output values, and the library titer; (**b**) ELISA characterization of phages from different rounds of selection (10^11^ phages/well) binding to BoNT/A or BoNT/A-DTT (dithiothreitol) (100 ng/well). Triplicate measurements were averaged; (**c**) In vivo neutralizing potency and specificity of the polyclonal phage libraries obtained in the second round of selection on BoNT/A and BoNT/A-DTT.

To generate a panel of single-domain antibodies to BoNT/A, we constructed an alpaca immune VHH library for display on the bacteriophage surface by cloning the nucleotide sequences coding for VHH repertoires of the immunized alpaca into an expression phagemid vector. After the transformation of recombinant phagemids into competent *E. coli* cells, a library, with a size of 5 × 10^6^ phagemids, was obtained. Phage display and two rounds of selection were performed for acquiring phage libraries. Purified BoNT/A was used as an antigen for the first round. Two independent second rounds were performed separately, with BoNT/A and BoNT/A-DTT used as antigens. DTT reduction was used for fragmenting toxins into two constituent parts—the heavy chain (HC) and light chain (LC) [34]–for more uniform absorption of chains on the immunoplate’s surface and to evenly distribute the epitopes on both chains. The polyclonal phage library titer for all rounds was approximately 10^13^ CFU (colony-forming unit)/mL (Figure 2a).

After two rounds of selection, the specificity of each library was determined by ELISA (Figure 2b). To test the neutralizing potency and specificity of the polyclonal phage libraries obtained in the second round of selection, an in vivo toxin neutralization assay was performed (Figure 2c). BALB/c female mice were divided into groups and received one intraperitoneal injection with 10 LD_50_ or 50 LD_50_ BoNT/A after 1 h incubation with phage libraries. Previously obtained phages specific to tetanus neurotoxin (TeNT) were used for controls as non-specific phages. All mice in the positive control group survived, while all mice in the negative control group and the TeNT group died. Challenging two experimental groups with 10 LD_50_ and 50 LD_50_ provided full and partial protection, thus allowing further selection of monoclones.

### 2.3. Selection of Individual VHH Clones and In Vivo Monoclonal Neutralization Assay

**Figure 3:**
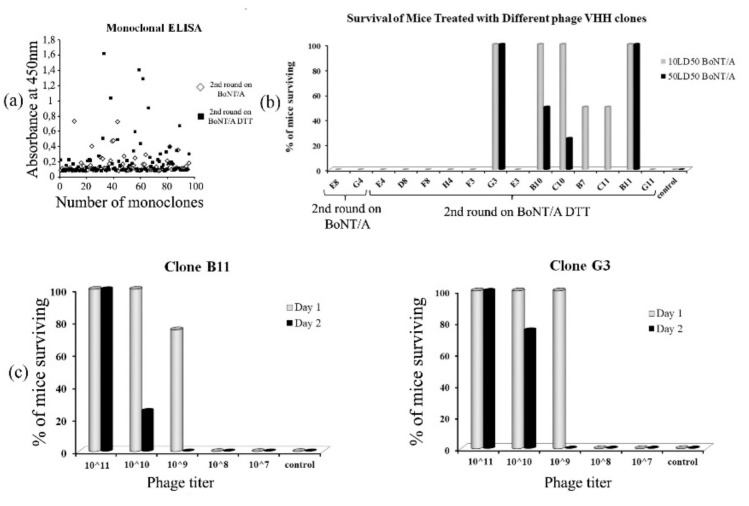
(**a**) Phage ELISA, showing binding of selected VHHs to BoNT/A and BoNT/A-DTT; (**b**) In vivo neutralizing potency of the selected clones. Survival of mice challenged with different LD_50_ premixed with different phage VHH clones. White–10 LD_50_, black–50 LD_50_; (**c**) Mouse survival rate when 10 LD_50_ BoNT/A was introduced mixed with the total colony-forming unit (CFU) different phage titers.

A total of 39 clones showing ELISA readouts higher than 0.2 (Figure 3a) and negligible reactivity with bovine serum albumin (BSA) were selected for sequencing. A total of 15 clones with different CDR3 amino acid sequences were selected for further research.

To test the neutralizing potency of the selected clones, an in vivo monoclonal phage neutralization assay was performed in which mice were administered the corresponding phage clone (Figure 3b). Each group received one intraperitoneal injection with 10 LD_50_ BoNT/A previously mixed with the corresponding phage clone at 10^11^ CFU. Only the four clones (B10, C10, B11, G3) that fully protected the mice against a 10 LD_50_ challenge were chosen to test their protectiveness with a 50 LD_50_ challenge. Mouse groups, which received clones B10 and C10 premixed with BoNT/A, were partially protected, while two other groups, which received clones B11 and G3 premixed with BoNT/A, showed 100% protection. Thus, clones B11 and G3 were chosen for further research as the most protective. It should be noted that the clones obtained by selection on BoNT/A-DTT showed a greater diversity in the CDR3 sequence and demonstrated neutralizing activity, unlike the clones, which were selected on BoNT/A.

The most protective clones, B11 and G3, were titrated in series down to 10^7^ CFU and introduced intraperitoneally into mice simultaneously with 10 LD_50_ of the toxin. The neutralizing potency of both clones was comparable, with the lower threshold being 10^10^ CFU (Figure 3c).

Finally, both clones were tested for cross-reactivity protectiveness with another toxin serotype, BoNT/B. Each group of mice was challenged with 5 LD_50_ of the toxin and administered 10^11^ CFU/mL phages. Both clones failed to protect the mice, demonstrating that the two protective clones are specific to BoNT/A.

### 2.4. Modification of VHH Clones to Improve their Protective Activity

**Figure 4:**
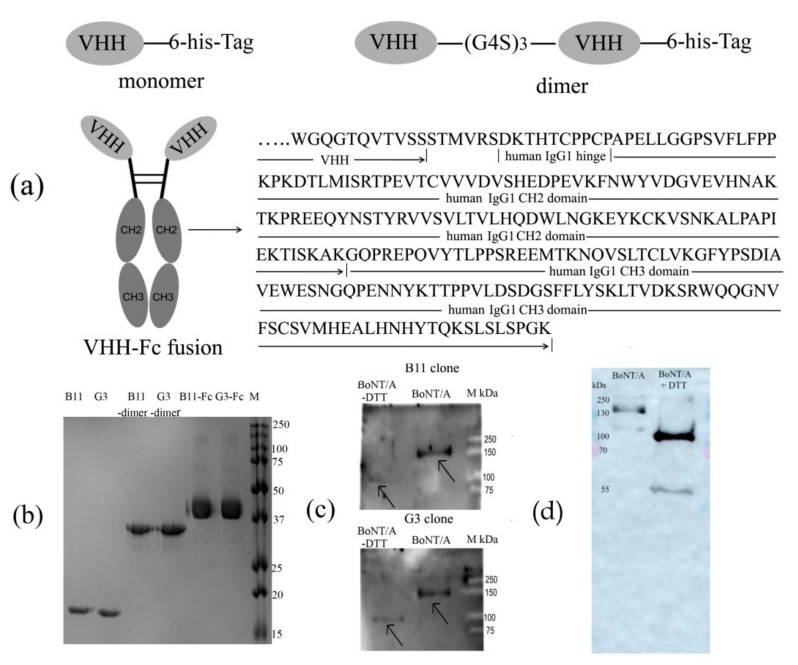
(**a**) Diagram of the VHHs monomer, VHHs dimer with (Gly4Ser)_3_ linker, and VHHs fused with human Fc (fragment crystallizable) fragments with the sequences of the hinge region and the Fc-fragment; (**b**) SDS-PAGE of the purified VHHs monomer, VHHs dimer with (Gly4Ser)_3_ linker, and VHH-Fc in 15% gel after staining with Coomassie blue. B11, G3, B11-d, G3-d, B11-Fc, G3-Fc – lanes with samples, M – dual-color molecular marker; (**c**) Immunoblot (under reducing conditions) of BoNT/A and BoNT/A-DTT with specific antibodies, B11-Fc and G3-Fc, with anti-human IgG (Fc-specific)-peroxidase antibodies (1:2500) used as detection reagent. M – dual-color molecular marker. A total of 500 ng of the toxin was used per well. BoNT/A-DTT preparations contained approximately 250 ng of the toxin from the dilution by the DTT reagent; (**d**) Immunoblot of BoNT/A (500 ng) and BoNT/A-DTT (500 ng under reducing conditions) with rabbit antibodies to BoNT/A (1:1000; Gamaleya’s Clostridiosis Laboratory, Moscow, Russia) and GAR-HRP (Goat Anti-Rabbit Horseradish Peroxidase 1:5000; Bio-Rad, Hercules, CA, USA).

To increase the protectiveness of the selected clones by increasing the antibody circulation time in the blood and/or their additional functionality, two modified constructions were synthesized. One construction was a dimer form of each clone (B11-dimer and G3-dimer) held by a glycine-serine linker (Gly4Ser)_3_ and expressed in the pET30 plasmid. The second construction was a monomer of each clone linked to a human IgG Fc fragment (B11-Fc and G3-Fc) (Figure 4a). Fc-modifications have been used to increase the antibody circulation time in blood. Human Fc-fragments cross-react with murine Fc-receptors and are capable of binding mouse FcRn [35,36]. Human Fc was chosen for these constructions to be used in further research and clinics. The dimers were produced and purified from the bacterial periplasmic fraction and verified by SDS-PAGE to be ~30 kDa. VHHs fused with Fc fragments were produced in Chinese hamster ovary (CHO-S) cells and verified by SDS-PAGE to be ~40 kDa under reducing conditions (Figure 4b). To determine the polypeptide chain of the toxin that bound specific antibodies, it was denatured by DTT into its HC and LC. SDS-PAGE was performed in denaturing conditions followed by western blot. B11-Fc and G3-Fc clones were tested, and anti-human IgG (Fc-specific)-peroxidase antibodies (1:2500) were used as detection reagents (Figure 4c). These antibodies were associated with the HC (100 kDa) of the toxin, which corresponds to the receptor domain. An immunoblot with BoNT/A and BoNT/A-DTT (under reducing conditions) were performed as control (Figure 4d). We investigated the affinity of clones B11 and G3 via surface plasmon resonance (SPR). Amine coupling was used to immobilize BoNT/A and BoNT/A-DTT on the sensor chip. The kinetic binding on- and off-rates between the antibodies and the toxin were determined from sensorgram analysis and used to calculate the equilibrium dissociation constant (KD) (Table 1).

**Figure 5:**
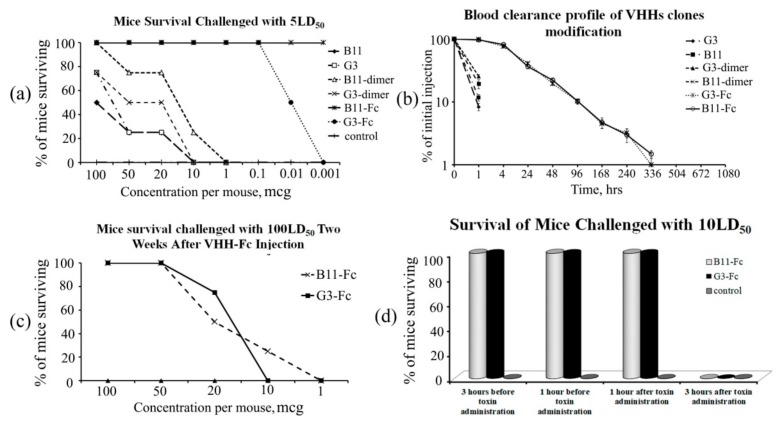
(**a**) Mice survival when challenged with lethal dose (5 LD_50_) premixed with VHHs monomers, VHHs dimers, or VHHs fused with Fc fragments. The appropriate amount of antibodies was mixed with 5 LD_50_ of BoNT/A for one hour and injected intraperitoneally. The final number of surviving mice was determined four days later; (**b**) Blood clearance profile of G3, B11, G3-dimer, B11-dimer, G3-Fc, or B11-Fc in BALB/c mice. After intravenous (i.v.) injection of 100 μg of different antibody constructs into the tail vein of the mice, the concentrations of the molecules in the sera at indicated time points were measured by ELISA; (**c**) Survival of mice administered with intraperitoneal 100 LD_50_ two weeks after antibody injection; (**d**) Survival of mice administered with B11-Fc and G3-Fc one hour and three hours before and after toxin challenge with intraperitoneal 10 LD_50_.

For the in vivo protection analysis, modified forms of VHHs, different amounts of monomers, dimers, and VHHs fused with Fc fragments were premixed with 5 LD_50_ BoNT/A and injected intraperitoneally into mice (Figure 5a). Monomers had only partial protectiveness even at the highest amount, 100 μg, with protectiveness gradually decreasing and failing at 10 μg. The dimers showed a better result on average, with full protectiveness of the B11-dimer at 100 μg and partial protectiveness at lower amounts, failing at 1 μg, as well as partial protectiveness of the G3-dimer at 100–20 μg amounts, failing at 10 μg. The best results were demonstrated by VHHs fused with Fc fragments, with G3-Fc failing to fully protect the mice at 0.1 μg and B11-Fc protecting the mice at amounts as low as 0.001 μg. All forms of VHHs partially protected the mice at high amounts, whereas VHHs fused with Fc fragments could protect the mice even at the lowest amounts tested (0.1–0.001 μg).

To assess the circulation time of various antibody modifications in the blood after a single injection, ELISA was used to measure the concentrations of G3, B11, G3-dimer, B11-dimer, G3-Fc, or B11-Fc in mouse blood taken at different time points after injection. The concentrations of G3, B11, as well as B11-dimer and G3-dimer, detected at 1 h post-injection were only about 10% of the initial level observed at 0 h. However, B11-Fc and G3-Fc modification constructs had a relatively long serum half-life. The concentrations of B11-Fc and G3-Fc 96 h post-injection were approximately 10% of the initial level. Moreover, two weeks after injection, the concentrations of B11-Fc and G3-Fc antibodies were approximately 1% of the initial level. These data are in agreement with reports on other VHHs with monomeric and fusionbody formats [37] (Figure 5b).

VHHs fused with Fc fragments demonstrated the best protection, and the mice treated with these preparations were alive two weeks after the end of the experiment. Based on the analysis of B11-Fc and G3-Fc clones’ circulation time in the serum (presence of antibodies 14 days after injection), we decided to conduct an experiment on the survival of these mice, which previously received a single injection of the VHHs with the Fc fragment, with a repeated administration of only the lethal toxin dose 14 days after the original administration. These mice were challenged with BoNT/A 100 LD_50_ to examine their protection over time (Figure 5c). All mice that previously received 100–50 μg of VHH-Fc were still fully protected. The mice which previously received 20–10 μg of the VHH-Fc were partially protected (75–25%) (clone B11-Fc) or not (clone G3-Fc). After two weeks, 1 μg of the preparation had no protectiveness.

We also tested the prophylactic efficacy and possible treatment of the toxin by administering the two selected Fc-fused clones one hour and three hours before and after toxin challenge with 10 LD_50_ (Figure 5d). Both clones fully protected the mice before the toxin challenge and one hour after. Three hours after toxin administration, the clones lacked protectiveness.

Overall, we obtained numerous clones after two rounds of biopanning; we selected 15 clones for initial analysis based on their CDR3s, chose two clones (B11 and G3) with the best pre-mixed results in phage form in vivo, produced them in protein form, and modified their structure and characteristics by dimerization via a (Gly4Ser)_3_ linker and fusion to a human IgG Fc fragment to enhance their protective activity.

We demonstrated that modification of neutralizing VHHs with an Fc fragment (fusionbody) significantly increased the circulation time of antibodies in the blood. At the same time, Fc fusion significantly increased the protective activity of VHH clones to more than 1000 times compared to monomeric forms. Moreover, clone B11-Fc increased the protective activity at least 100,000 times compared to the monomeric form, with 100% protectiveness even at 0.001 μg. The estimated molecular ratio of the 5 LD_50_ to 0.001 μg pre-mix was 1:22.

## 3. Discussion

BoNT is the most potent and lethal known toxin. Therefore, the development of new therapeutic agents for exposure prevention and treatment is essential. Therapeutic approaches have been summarized in previous publications [11]. Along with serum therapy, specific antibodies are a promising tool for neutralizing BoNTs. Approaches for the generation, selection, combination, and modification of mAbs against BoNT/A have been described and tested [38,39,40,41,42].

Camelid VHHs are a popular tool for constructing recombinant antibodies to detect and neutralize a range of targets [18,19,20,21,22,23,24]. In particular, it has been shown that HC-only antibodies or VHHs derived from HC-only antibodies, produced by camelids demonstrate strong anti-BoNT activities in animal models [6,43,44]. The main differences of VHHs from conventional antibodies include specific conserved amino acid substitutions in framework region 2 (FR2) that make contact with the LC of classical antibodies, as well as a longer length, more variable amino acid composition in the CDR3s, and a folded back loop [27,45,46]. Specific VHHs can be isolated from VHH libraries. One type of such libraries—immune libraries—can be produced based on peripheral blood lymphocytes isolated from camelids that have been immunized with an antigen of interest in a prime-boost strategy [47]. In our study, we used a toxoid consisting of BoNT/A toxin and small amounts of hemagglutinin (HA) as an antigen and screened for VHHs against BoNT/A and BoNT/A-DTT using phage display technology.

The neutralizing clones in our work were selected on BoNT/A-DTT. It should be noted that the neutralizing clones were later found in the BoNT/A library as well. However, there were fewer of them, so a greater number of clones had to be analyzed. Overall, approximately 300 clones from BoNT/A-DTT and more than 1000 clones from BoNT/A libraries were examined. The approach of splitting the toxin into its HC and LC enriches the neutralizing clones in the library. Based on their neutralizing activity when pre-mixed with different toxin LD_50_ doses, two clones were selected. Interestingly, all clones with neutralizing potency did not have the highest ELISA signals. Therefore, the OD (optical density) of the ELISA signal does not always mean high neutralizing activity and in vivo protective activity of these VHHs.

The next step was to produce and purify VHHs in protein form to test their neutralizing potency in vivo. However, their protection was weak. Even when pre-mixing a 100 μg dose of these VHHs with the toxin, they had a 50–75% protection efficiency. Administration of lower doses of the preparation was non-protective, with 10 μg lacking effectiveness. Therefore, two clones—B11 and G3—were modified to increase their half-life and protection efficacy. Increasing the VHH size by oligomerization—the coupling of two or more VHHs via a specific linker—increased their protectiveness; however, even bivalent constructs were rapidly cleared [46].

Another approach was VHH fusion to an Fc region of the IgG molecule. Constructs with fused Fc fragments were previously made to extend the antibody half-life to neutralize the Middle East respiratory syndrome coronavirus (MERS-CoV) [48,49], target the C-X-C chemokine receptor type 4 (CXCR4) to prevent human immunodeficiency virus 1 (HIV-1) strain entry and replication in vitro [50], and increase the Fc effector functions to neutralize rotavirus [51] as well as the influenza virus HA [52]. A recent study of modified VHHs dimers and VHHs fused to Fcs targeting unique epitopes on the immunogen, composed of a portion of the central delivery domain and the entire combined repetitive oligopeptides (CROPs) domain of *Clostridium difficile* type B, showed modest and much greater toxin inhibitions, respectively [53]. It should be noted that a combined approach for efficient serum clearance of BoNT/A has been developed before. Sepulveda et al. [54] used single-chain variable fragments (scFvs) as protein binding agents pre-complexed with one or two single anti-tag mAbs with an Fc domain and tested this construction in vivo for protection and pharmacokinetics. When three or four types of such constructions were given simultaneously, mice were protected at high LD_50_s, and BoNT/A was rapidly cleared from the sera. Protection against BoNT/A light chain was observed when scFvs fused with Fc fragments (scFv-Fc) obtained from a macaque immune library were tested ex vivo [55]. Furthermore, the substantial contribution of the homologous Fc fragment to the potency of three individual anti-botulinum mAbs in antibody preparations has been demonstrated [56].

In our work, two chosen clones were dimerized via a (Gly4Ser)_3_ linker or fusion to human Fc fragments to stabilize the molecules and slow down their clearance. These modifications led to improved protectiveness of the preparations. The dimers demonstrated protectiveness at lower doses compared to VHHs, with 75–50% protection efficiency at 50 and 20 μg. Clone G3-dimer failed to protect the mice at 10 μg, like the VHHs, while clone B11-dimer showed partial protectiveness and failed at 1 μg. Therefore, the effective dose (ED_50_) for clone B11-dimer was 15 μg, and for the clone G3-dimer, was 20 μg.

The best results were obtained after the fusion of the VHHs to Fc fragments, which is consistent with previous research. Both clones fully protected the mice at doses as low as 0.1 μg. The protection provided by G3-Fc was half at 0.01 μg, and the clone failed to protect the mice at 0.001 μg. B11-Fc demonstrated full protection down to the lowest tested dose of 0.001 μg. The dose at which its protectiveness begins to decrease was not reached. Therefore, both clones with Fc fragments showed at least a 1000-fold improvement in protectiveness compared to conventional VHHs and dimers. The ED50 dose for clone B11-Fc could not be determined, while it was 0.01 μg for clone G3-Fc.

Besides, the pharmacokinetics analysis of various antibody forms showed that clones B11 and G3 containing the IgG Fc fragment were detected in the mouse serum 14 days after a single injection, which is confirmed by full or partial animal protection from the challenge with the toxin 14 days after administration of various B11-Fc and G3-Fc doses.

Testing the prophylaxis and possible treatment of the toxin by administering B11-Fc and G3-Fc one hour and three hours before and after toxin challenge with 10 LD_50_ showed that both clones fully protected the mice before the toxin challenge as well as one hour after, but failed to protect the mice three hours after toxin administration, suggesting that these clones could be used for prophylaxis and emergency therapy immediately after toxin administration. This corresponds with the results obtained previously by Sepulveda et al. [54] showing that mice receiving scFvs pre-complexed with anti-tagged mAbs with Fc fragments were protected only two hours after intoxication, while four hours after, toxin administration lethality was only delayed.

The acquisition of antibodies against BoNT/A and confirmation of their neutralizing potency in vivo in phage forms when pre-mixed with the toxin has provided a new method to screen for neutralizing VHHs before obtaining them in protein form, which can efficiently reduce time and material costs for their production and testing. VHHs, along with their modifications as oligomers and fusions to Fc fragments of the IgG, increase the range of options for BoNT/A targeting and neutralization by improving the blood circulation time and therapeutic potential.

## 4. Materials and Methods

### 4.1. Ethics Statement

The experimental procedures conformed to the Guide for the Care and Use of Laboratory Animals published by the National Institutes of Health (NIH Publication #85–23, revised 1996) and National Standard of the Russian Federation GOST R 53434–2009, approved by the Institutional Animal Care and Use Committee (IACUC) of the Federal Research Centre of Epidemiology and Microbiology named after Honorary Academician N.F. Gamaleya and were performed under Protocol #16 from 8 February 2019. All persons using or caring for animals in research underwent yearly training as required by the IACUC.

### 4.2. Animal Housing Conditions

Alpaca immunization and blood collection were performed on one clinically healthy 4-year-old male alpaca (*Vicugna pacos*) on the “Russian Alpacas” Farm, private land located in Pokhodkino, Moscow Region, Russia. This sample collection did not involve endangered or protected species, and no specific permissions were required for these locations/activities.

Six-week-old female Balb/c mice (weighing 18–20 g) were purchased from “Pushchino breeding facility” (Pushchino, Moscow Region, Russia) accredited by Association for Assessment and Accreditation of Laboratory Animal Care (AAALAC International) and maintained at the central animal facility at the Gamaleya Research Center of Epidemiology and Microbiology. Mice were kept at a constant temperature (22 ± 2 °C) and relative humidity (50%) with 12 h of artificial light per day. They were housed in individual cages (8 per cage). Mice were fed with dried food and water ad libitum. Mice were observed every two hours post-injection except during the night for one week. The animals with characteristic symptoms of botulism, including muscle paralysis and respiratory difficulty, were euthanized by cervical dislocation.

### 4.3. Antigen Preparation

BoNT/A from the *C. botulinum* strain A98 was obtained from the Gamaleya Research Center, Moscow, Russia collection. BoNT/A is very toxic; therefore, appropriate safety precautions were taken during these experiments. The neurotoxin was handled at a Class 2 biosafety cabinet. The antigen was a toxoid consisting of BoNT/A toxin and small amounts of HA, and 0.1% formaldehyde was used for toxoid preparation. The treatment was carried out for seven days at 40 °C [57]. Mice received intraperitoneal injections to determine residual toxicity, which showed its absence. Before use in alpacas, the antigen was purified through a 0.22 μm filter. The final concentration was 60 μg/mL. Before immunization, the neurotoxin was inactivated by 0.05% formaldehyde at pH = 6 during 3–5 days. BoNT/A preparations were used with Freund’s adjuvant without pre-adsorption.

### 4.4. Alpaca Immunization

Alpaca immunization was performed using five sequential injections with an interval of 14 days between the first and second immunizations and 10 days between all subsequent immunizations. For the first injection time, 60 μg (1 mL) of the antigen and complete Freund’s adjuvant (Sigma, St. Louis, MO, USA) at the v/v ratio of 1:1 were administered. The four subsequent immunizations were performed with 90 μg (1.5 mL) of the antigen and incomplete Freund’s adjuvant (Sigma, St. Louis, MO, USA) at the v/v ratio of 1:1. Small blood samples (5–7 mL) were collected before immunization, as well as after the third and fifth immunizations as a control. Five days after the final injection, 50 mL blood sample was collected and placed into a sterile vacuum collection tube with lithium heparin to prevent blood clotting.

### 4.5. Phage Display Library Construction

mRNA isolation, PCR amplification, and library construction were performed, as described elsewhere [58,59]. Briefly, camelid VHHs from peripheral blood B-lymphocytes (about 10^6^ cells/mL) of the immunized alpaca were cloned into a pHEN1 expression phagemid vector [60]. The primer set used for PCR amplification of antibody genes appended the SfiI (NEB) restriction site at the 5′-end and the NotI (NEB) site at its 3′-end (Table 2). Recombinant phagemids were introduced into freshly prepared competent suppressor TG1 *E. coli* cells (Lucigen, Middleton, WI, USA). Using this method, a library of 5 × 10^6^ individual clones for biopanning and isolation was obtained.

### 4.6. Phage Preparation and Biopanning

The bacteria from the VHH library were added to 2 × YT medium (Sigma, St. Louis, MO, USA) (with 100 μg/mL ampicillin and 1% glucose) and incubated at 37 °C in a culture shaker at 210 rpm to an OD600 = 0.6. KM13 helper phages (Patrick Chames “Antibody therapeutics and Immunotargeting team” of the Cancer Research Center of Marseille) were added to the bacteria (multiplicity of infection, MOI = 20) and left without shaking at 37 °C for 30 min. The culture was centrifuged at 4000 rpm for 20 min at 4 °C, and the cell pellets were resuspended in 2 × YT medium (with 100 μg/mL ampicillin and 50 μg/mL kanamycin) and cultured overnight at 30 °C, in a culture shaker at 210 rpm. The next day, the culture was centrifuged, the supernatant was purified and concentrated by 20% polyethylene glycol (PEG) 8000, 2.5 M NaCl precipitation, and the pellet was resuspended in phosphate-buffered saline (PBS) with 80% glycerol.

BoNT/A-DTT was treated with 1 mM DTT for at least 30 min at 25 °C. Microtiter plate wells were coated with 5 μg of BoNT/A for the first round and with BoNT/A and BoNT/A-DTT for the second round in 0.05 M NaHCO_3_ buffer (pH = 9.6) at 4 °C overnight. After rinsing three times with PBS with 0.1% Tween 20 (TPBS), the plate was blocked with blocking buffer (TPBS with 5% non-fat dried milk) at 37 °C for 1 h. A total of ~10^11^ phages were added to each well and incubated at 37 °C for 1 h. Unbound phages were removed by washing 10 times with TPBS. The bound phages were eluted by trypsin with a final concentration of 1 mg/mL. TG1 *E. coli* cells at OD600 = 0.6 were infected with the eluted phages and incubated without shaking at 37 °C for 30 min. After culturing the mixture in 2 × YT agar plates (with 100 μg/mL ampicillin and 1% glucose) at 37 °C overnight, the cells were scraped. Recombinant phages were obtained by packaging with KM13 helper phages, and their titers were determined. A total of two panning rounds were performed.

### 4.7. ELISA Screening for Specific VHHs

For a polyclonal ELISA, an immunoplate (MaxiSorp, Nunc) was coated with 100 ng of BoNT/A and BoNT/A-DTT with 0.05 M NaHCO_3_ buffer (pH = 9.6) at 4 °C overnight. The plate was rinsed three times with TPBS, blocked with blocking buffer at 37 °C for 1 h, and a total of ~10^11^ phages from the starting library or the first or second rounds were added to each well and incubated at 37 °C for 1 h. The unbound phages were removed by washing 10 times with TPBS. The plate wells were detected by horseradish peroxidase (HRP)-conjugated anti-M13 antibodies (1:5000) (Abcam, Cambridge, UK), followed by the addition of peroxidase substrate 3,3′,5,5′-tetramethylbenzidine (TMB) (Bio-Rad, Hercules, CA, USA). The reaction was stopped by 1 M H2SO4, and the absorbance at 450 nm was read with an iEMS Reader MF (Thermo Labsystems, Waltham, MA, USA). A monoclonal ELISA was performed following the same protocol, with particular clones bound to BoNT/A and BoNT/A-DTT being selected, and the phage plasmids were isolated for sequencing. Antibody genes in phagemids were sequenced with a primer set used for PCR according to the protocol of Big Dye Terminator 3.1 Cycle Sequencing kit for the Genetic Analyzer 3500 Applied Biosystems (Waltham, MA, USA). The electrophoretic DNA separation was performed in 50 cm capillaries with POP7 polymer.

### 4.8. Protein Expression and Purification

The selected VHHs expressed in pHEN1 plasmid were transformed into *E. coli* BL21 cells (NEB, Ipswich, MA, USA) for expression and purification. An overnight culture was obtained at 37 °C, in a culture shaker at 210 rpm. The next day, the cells were harvested by centrifugation, lysed by BugBuster Protein Extraction Reagent (Novagen, Madison, WI, USA), and VHHs were purified from the lysate by TALON Superflow containing Co^2+^ agarose (GE Healthcare Bio-Sciences AB, Uppsala, Sweden). The eluted fraction was subjected to dialysis with Visking dialysis tubing (MWCO 12000–14000) (Serva, Heidelberg, Germany). VHHs were separated by SDS-PAGE (Bio-Rad, Hercules, CA, USA) under denaturing conditions and had an expected molecular weight of ~15 kDa.

### 4.9. Affinity and Binding Kinetic Measurements

Antibody affinity was determined by surface plasmon resonance (SPR) using a Biacore 3000 instrument (GE Healthcare Bio-Sciences AB, Uppsala, Sweden). BoNT/A and BoNT/A-DTT were immobilized on the surface of CM5 sensor chips in the amount of 10 μg each in 10 mM sodium acetate buffer pH = 4.5 using the amine coupling kit recommended by the manufacturer (GE Healthcare Bio-Sciences AB, Uppsala, Sweden). VHHs (2-fold dilutions from 300 μg down to 0 μg) were captured on the sensor chips and submitted at a constant flow rate of 15 μL/min with HBS-EP (0.01 M HEPES pH 7.4, 0.15 M NaCl, 3 mM EDTA, 0.005% v/v Surfactant P20) as a running buffer at 25 °C with an injection time of 3 min and dissociation time of 10 min. After each injection, the chip surface was regenerated with 20 mM Tris-HCl, pH = 2.0 for 30 s at a flow rate of 20 μL/min. Calculations were performed using BIAEvaluation software (GE Healthcare Bio-Sciences AB, Uppsala, Sweden) with reference-subtracted fitting.

### 4.10. Production of VHHs in Dimer Form and Fused with IgG Fc Fragments

The selected VHHs were held by a glycine-serine linker (Gly4Ser)_3_ and expressed in the pET30 plasmid. Protein expression and purification were performed as for VHHs.

Nucleotide sequences of VHH genes fused to the human IgG Fc-fragment were synthesized and cloned into the plasmid pShuttle-CMVFUSE (Stratagene, La Jolla, CA, USA). The CHO-S cell culture (ThermoFisher, Waltham, MA, USA, R80007) was transiently transfected with pFUSE plasmid using the CHO Gro System (Mirus Bio, Madison, WI, USA), according to the manufacturer’s protocol. A plasmid carrying the green fluorescent protein gene in a 10% amount was used as control. The efficiency of transfection was assessed using Axio Imager Z1 (Carl Zeiss, Oberkochen, Germany) and determined by the number of fluorescence cells compared to their overall amount, which was 90%. Cells were cultured in shake flasks at 125 rpm, 5% CO_2_, 80% humidity, at 37 °C during transfection and 32 °C 24 h after the transfection for 10 days. Starting from day three, Cellboosts 7a (3%), 7b (0.3%) (HyClone, San Angeo, TX, USA), and 1% Sigma Bioreactor Feed (Sigma, St. Louis, MO, USA) were added each day. After 10 days of cultivation, the culture was clarified by centrifugation at 5000× *g* and cleared through a 0.8 μm filter. The antibodies were purified using protein A affinity chromatography on an AKTA start chromatography system (GE Healthcare Bio-Sciences AB, Uppsala, Sweden), with a mAbSelect SuRe 1 mL column (GE Healthcare Bio-Sciences AB, Uppsala, Sweden), according to the manufacturer’s protocol.

### 4.11. Identification of the Toxin Polypeptide Chain that Binds Antibodies by Western Blot

To determine which BoNT/A polypeptide chain binds VHHs, the toxin was denatured by DTT into its HC and LC. SDS-PAGE was performed using a mini-protean TGX stain-free precast gel (Bio-Rad, Hercules, CA, USA) in denaturing conditions. The separated bands were transferred onto a nitrocellulose membrane using a Trans-Blot Turbo System (Bio-Rad, Hercules, CA, USA). The membrane was blocked with a blocking buffer at 37 °C for 1 h. VHHs were diluted in the blocking buffer, added to the membrane, and incubated at 37 °C for 1 h. After rinsing the membrane three times with TPBS, anti-His-tag-HRP-mouse antibodies (GenScript, Piscataway, NJ, USA) diluted 1:5000 in blocking buffer were added, and the membrane was then incubated at 37 °C for 1 h. The membrane was rinsed three times with TPBS, followed by the addition of Clarity Western ECL Blotting Substrates (Bio-Rad, Hercules, CA, USA) for detection. The membrane was visualized on an Amersham Imager 600 (GE Healthcare, Buckinghamshire, UK).

### 4.12. Toxin Preparation for In Vivo Neutralization Assay

The neurotoxin as a multi-oligomeric complex toxin with HAs and NTNHA (non-toxic non-hemagglutinin) (800 kDa) was used for immunization. Toxins were prepared and purified as follows. The strain was cultivated under anaerobic conditions for five days. Bacterial cells were separated by centrifugation at 5000× *g* for 30 min at 10 °C. The proteins from the culture filtrate were concentrated by acid precipitation at pH = 3.8 for 45 min. The precipitate was separated by centrifugation at 12,000× *g* for 30 min at 10 °C and dissolved in 47 mM citrate-phosphate buffer with pH = 5.6. Gel filtration S300 and ion-exchange chromatography on AKTA start chromatography system (GE Healthcare Bio-Sciences AB, Uppsala, Sweden) on DE cellulose (Pharmacia, Uppsala, Sweden) were then carried out. The toxin (90–95%, 150 kDa) was purified by additional DE cellulose chromatography (Pharmacia, Uppsala, Sweden) in borate buffer pH = 8 and eluted by 50 mM NaCl. Non-sorbed material containing specific antigenic and biological activity was used as BoNT complex with HA. The specific antigenic activity was a positive reaction with monospecific antibodies to BoNT/A, HA, and NTNHA (Gamaleya Laboratory of Clostridiosis and commercial preparation of Scientific Centre for Expert Evaluation of Medicinal Products Russian Federation).

### 4.13. In Vivo Toxin Neutralization with Phages or Proteins

Phages with titers 10^11^–10^12^ CFU/mL were prepared, as described above, and mixed with standard saline solution. VHH proteins at different amounts ranging from 100 μg to 0.001 μg were prepared, as described above, and mixed with standard saline solution. Balb/c 18–20 g female mice were divided into groups of four, including the positive and negative control groups. Phages or VHH proteins were premixed with the appropriate toxin LD_50_ (1LD ~ 30 pg BoNT/A) and incubated for 1 h at 37 °C. All mice received one intraperitoneal 500 μL injection of phages or proteins premixed with various LD_50_s of the toxin and were observed once a day for one week. A specific pathological pattern was observed in sick mice, which is the pathological pattern with dystonia’s abdominal muscles (waistline increasing) and death at 30–50 pg that is neutralized by monospecific antibodies to BoNT/A. The positive control group received the rabbit antitoxin IgG (Gamaleya Laboratory of Clostridiosis), and the negative control group received a standard saline solution.

### 4.14. Blood Clearance of VHHs Modifications in Mice

A group of five six-week-old female Balb/c mice was intravenously (i.v.) injected with 100 μg G3, B11, G3-dimer, B11-dimer, G3-Fc, or B11-Fc into the tail vein. Blood was collected from the facial vein at 0, 1, 4, 24, 48, 96, 168, 240, 336 h time points. Sera were separated and stored at −20 °C until further use. Concentrations of the injected antibody molecules in the above-collected samples were measured by ELISA. For ELISA, BoNT/A was coated on microtiter plates (Nunc) overnight at 4 °C at 100 ng/well in 50 mM bicarbonate buffer. After washing three times with TPBS, plates were blocked with 5% dry milk in PBS for one hour at 37 °C. Then, 200× diluted sera were added to the wells, followed by a one-hour incubation. Mouse anti-His-tag antibody [HRP] (1:1000) (GenScript, Piscataway, NJ, USA) was used to detect monomers and dimers of G3 and B11 antibodies in the mouse sera. Goat anti-human IgG (Fc-specific) antibody [HRP] (1:2000) (Merck, Kenilworth, NJ, USA) was used to detect G3-Fc and B11-Fc antibodies in the mouse sera. Serial dilutions of BoNT/A in mouse serum were used to make a standard curve for antibody concentration analysis using computer software ELISA Master (AlkorBio, St. Petersburg, Russia).

## Figures and Tables

**Table 1:** Kinetic parameters of antibody interactions with the toxin obtained by SPR (surface plasmon resonance). Association (on-rate, K_a_), dissociation (off-rate, K_d_), maximum analyte binding capacity (R_max_), equilibrium association constants (K_A_), equilibrium dissociation constants (K_D_), and Chi^2^ for the chosen VHHs (variable domains of heavy-chain only antibodies) binding to botulinum neurotoxin (BoNT)/A or BoNT/A-DTT (dithiothreitol).

Clones Binding to BoNT/A-DTT	K_a_ (1/Ms)	K_d_ (1/s)	R_max_ (RU)	K_A_ (1/M)	K_D_ (M)	Chi^2^
B11 (BoNT/A)	8.67 × 10^3^	2.3 × 10^−4^	17	3.37 × 10^7^	2.65× 10^−8^	3.9
B11 (BoNT/A-DTT)	7.06 × 10^3^	4.37 × 10^−4^	23.6	1.62 × 10^7^	6.19 × 10^−8^	1.8
G3 (BoNT/A)	1.51 × 10^4^	4.54 × 10^−4^	10.4	3.32 × 10^7^	3.01 × 10^−8^	1.0
G3 (BoNT/A-DTT)	1.72 × 10^4^	1.84 × 10^−3^	43.1	9.34 × 10^6^	1.07 × 10^−7^	5.7

**Table 2:** Primer sets used for first- and second-round PCR.

Primer	Sequence
VH1-SfiI	CATGCCATGACTCGCGGCCCAGCCGGCCATGGCCCAGGTGCAGCTGGTGCAGTCTGG
VH2-SfiI	CATGCCATGACTCGCGGCCCAGCCGGCCATGGCCCAGGTCACCTTGAAGGAGTCTGG
VH3-SfiI	CATGCCATGACTCGCGGCCCAGCCGGCCATGGCCGAGGTGCAGCTGGTGGAGTCTGG
VH4-SfiI	CATGCCATGACTCGCGGCCCAGCCGGCCATGGCCCAGGTGCAGCTGCAGGAGTCGGG
CH2FORTA4	CGCCATCAAGGTACCAGTTGA
VHH-NotI	CCACGATTCTGCGGCCGCTGAGGAGACRGTGACCTGGGTCC

## References

[1] Gill D. (1982). Bacterial toxins—A table of lethal amounts. Microbiol. Rev..

[2] Goonetilleke A., Harris J. (2004). Clostridial neurotoxins. J. Neurol. Neurosurg. Psychiatry.

[3] Arnon S., Schechter R., Inglesby T., Henderson D.A., Bartlett J.G., Ascher M.S., Eitzen E., Fine A.D., Hauer J., Layton M. (2001). Botulinum toxin as a biological weapon—Medical and public health management. JAMA.

[4] Emmeluth D. (2010). Botulism.

[5] Spickler A.R. (2018). Botulism.

[6] Yao G., Lam K.H., Weisemann J., Peng L., Krez N., Perry K., Shoemaker C.B., Dong M., Rummel A., Jin R. (2017). A camelid single-domain antibody neutralizes botulinum neurotoxin A by blocking host receptor binding. Sci. Rep..

[7] World Health Organization (2018). Botulism.

[8] Graham R., Thorp F. (1929). The effect of formalin on botulinum toxins A, B and C. J. Immunol..

[9] Centers for Disease Control and Prevention, Morbidity and Mortality Weekly Report (2011). Notice of CDC’s Discontinuation of Investigational Pentavalent (ABCDE) Botulinum Toxoid Vaccine for Workers at Risk for Occupational Exposure to Botulinum Toxins.

[10] Nantel A.J. (1999). Clostridium botulinum—International Programme on Chemical Safety—Bacteria.

[11] Patel K., Cai S., Singh B. (2014). Current strategies for designing antidotes against botulinum neurotoxins. Expert Opin. Drug. Discov..

[12] Yu R., Wang S., Yu Y.Z., Du W.S., Yang F., Yu W.Y., Sun Z.W. (2009). Neutralizing antibodies of botulinum neurotoxin serotype A screened from a fully synthetic human antibody phage display library. J. Biomol. Screen..

[13] Rasetti-Escargueil C., Avril A., Miethe S., Mazuet C., Derman Y., Selby K., Thullier P., Pelat T., Urbain R., Fontayne A. (2017). The European AntibotABE Framework Program and its update: Development of innovative botulinum antibodies. Toxins.

[14] Hamers-Casterman C., Atarhouch T., Muyldermans S., Robinson G., Hammers C., Bajyana Songa E., Bendahman N., Hammers R. (1993). Naturally-occurring antibodies devoid of light-chains. Nature.

[15] Harmsen M., De Haard H. (2007). Properties, production, and applications of camelid single-domain antibody fragments. Appl. Microbiol. Biotechnol..

[16] Wesolowski J., Alzogaray V., Reyelt J., Unger M., Juarez K., Urrutia M., Cauerhff A., Danquah W., Rissiek B., Scheuplein F. (2009). Single domain antibodies: Promising experimental and therapeutic tools in infection and immunity. Med. Microbiol. Immunol..

[17] Muyldermans S. (2013). Nanobodies: Natural single-domain antibodies. Ann. Rev. Biochem..

[18] Wu Y., Jiang S., Ying T. (2017). Single-Domain Antibodies as therapeutics against human viral diseases. Front. Immunol..

[19] Anderson G., Liu J., Hale M., Bernstein R.D., Moore M., Swain M.D., Goldman E.R. (2008). Development of antiricin single domain antibodies toward detection and therapeutic reagents. Anal. Chem..

[20] Burmistrova D., Tillib S., Shcheblyakov D., Dolzhikova I.D.V., Shcherbinin D.N., Zubkova O.V., Ivanova T.I., Tukhvatulin A.I., Shmarov M.M., Logunov D.Y. (2016). Genetic passive immunization with adenoviral vector expressing chimeric nanobody-Fc molecules as therapy for genital infection caused by *Mycoplasma hominis*. PLoS ONE.

[21] Rossotti M., Gonzalez-Techera A., Guarnaschelli J., Yim L., Camacho X., Fernández M., Cabral P., Leizagoyen C., Chabalgoity J.A., González-Sapienza G. (2015). Increasing the potency of neutralizing single-domain antibodies by functionalization with a CD11b/CD18 binding domain. MAbs.

[22] Unger M., Eichhoff A., Schumacher L., Strysio M., Menzel S., Schwan C., Alzogaray V., Zylberman V., Seman M., Brandner J. (2015). Selection of nanobodies that block the enzymatic and cytotoxic activities of the binary *Clostridium difficile* toxin CDT. Sci. Rep..

[23] Luiz M., Pereira S., Prado N., Gonçalves N.R., Kayano A.M., Moreira-Dill L.S., Sobrinho J.C., Zanchi F.B., Fuly A.L., Fernandes C.F. (2018). Camelid single-domain antibodies (VHHs) against Crotoxin: A basis for developing modular building blocks for the enhancement of treatment or diagnosis of crotalic envenoming. Toxins.

[24] Bernedo-Navarro R., Romao E., Yano T., Pinto J., De Greve H., Sterckx Y.G.J., Muyldermans S. (2018). Structural basis for the specific neutralization of Stx2a with a camelid single domain antibody fragment. Toxins.

[25] Dong J., Thompson A.A., Fan Y., Lou J., Conrad F., Ho M., Pires-Alves M., Wilson B.A., Stevens R.C., Marks J.D. (2010). A single-domain llama antibody potently inhibits the enzymatic activity of botulinum neurotoxin by binding to the non-catalytic α-exosite binding region. J. Mol. Biol..

[26] Thanongsaksrikul J., Chaicumpa W. (2011). Botulinum neurotoxins and botulism: A novel therapeutic approach. Toxins.

[27] Arbabi-Ghahroudi M. (2017). Camelid single-domain antibodies: Historical perspective and future outlook. Front. Immunol..

[28] (2019). CABLIVI.

[29] Jank L., Pinto-Espinoza C., Duan Y., Koch-Nolte F., Magnus T., Rissiek B. (2019). Current approaches and future perspectives for nanobodies in stroke diagnostic and therapy. Antibodies.

[30] Rotman M., Welling M.M., van den Boogaard M.L., Moursel L.G., van der Graaf L.M., van Buchem M.A., van der Maarel S.M., van der Weerd L. (2015). Fusion of hIgG1-Fc to 111In-anti-amyloid single domain antibody fragment VHH-pa2H prolongs blood residential time in APP/PS1 mice but does not increase brain uptake. Nucl. Med. Biol..

[31] Mukherjee J., Dmitriev I., Debatis M., Tremblay J.M., Beamer G., Kashentseva E.A., Curiel D.T., Shoemaker C.B. (2014). Prolonged prophylactic protection from botulism with a single adenovirus treatment promoting serum expression of a VHH-based antitoxin protein. PLoS ONE.

[32] Huang N.J., Pishesha N., Mukherjee J., Zhang S., Deshycka R., Sudaryo V., Dong M., Shoemaker C.B., Lodish H.F. (2017). Genetically engineered red cells expressing single domain camelid antibodies confer long-term protection against botulinum neurotoxin. Nat. Commun..

[33] Lu L., Suscovich T., Fortune S., Alter G. (2018). Beyond binding: Antibody effector functions in infectious diseases. Nat. Rev. Immunol..

[34] Pirazzini M., Rossetto O., Eleopra R., Montecucco C. (2017). Botulinum neurotoxins: Biology, pharmacology, and toxicology. Pharmacol. Rev..

[35] Abdiche Y.N., Yeung Y.A., Chaparro-Riggers J., Barman I., Strop P., Chin S.M., Pham A., Bolton G., McDonough D., Lindquist K. (2015). The neonatal Fc receptor (FcRn) binds independently to both sites of the IgG homodimer with identical affinity. MAbs.

[36] Derebe M.G., Nanjunda R.K., Gilliland G.L., Lacy E.R., Chiu M.L. (2018). Human IgG subclass cross-species reactivity to mouse and cynomolgus monkey Fcγ receptors. Immunol. Lett..

[37] Bell A., Wang Z.J., Arbabi-Ghahroudi M., Chang T.A., Durocher Y., Trojahn U., Baardsnes J., Jaramillo M.L., Li S., Baral T.N. (2010). Differential tumor-targeting abilities of three single-domain antibody formats. Cancer Lett..

[38] Wu H.C., Yen C.T., Huang Y.L., Tarn L.J., Lung C.C. (2001). Characterization of neutralizing antibodies and identification of neutralizing epitope mimics on the *Clostridium botulinum* neurotoxin type A. Appl. Environ. Microbiol..

[39] Nowakowski A., Wang C., Powers D., Amersdorfer P., Smith T.J., Montgomery V.A., Sheridan R., Blake R., Smith L.A., Marks J.D. (2002). Potent neutralization of botulinum neurotoxin by recombinant oligoclonal antibody. Proc. Natl. Acad. Sci. USA.

[40] Mazuet C., Dano J., Popoff M., Creminon C., Volland H. (2010). Characterization of botulinum neurotoxin type A neutralizing monoclonal antibodies and influence of their half-lives on therapeutic activity. PLoS ONE.

[41] Zhao H., Nakamura K., Kohda T., Mukamoto M., Kozaki S. (2012). Characterization of the monoclonal antibody response to botulinum neurotoxin type A in the complexed and uncomplexed forms. Jpn. J. Infect. Dis..

[42] Miethe S., Mazuet C., Liu Y., Tierney R., Rasetti-Escargueil C., Avril A., Frenzel A., Thullier P., Pelat T., Urbain R. (2016). Development of germline-humanized antibodies neutralizing botulinum neurotoxin A and B. PLoS ONE.

[43] Swain M.D., Anderson G.P., Zabetakis D., Bernstein R.D., Liu J.L., Sherwood L.J., Hayhurst A., Goldman E.R. (2010). Llama-derived single-domain antibodies for the detection of botulinum A neurotoxin. Anal. Bioanal. Chem..

[44] Tremblay J.M., Kuo C.L., Abeijon C., Sepulveda J., Oyler G., Hu X., Jin M.M., Shoemaker C.B. (2010). Camelid single domain antibodies (VHHs) as neuronal cell intrabody binding agents and inhibitors of *Clostridium botulinum* neurotoxin (BoNT) proteases. Toxicon.

[45] Henry K., MacKenzie C. (2018). Antigen recognition by single-domain antibodies: Structural latitudes and constraints. MAbs.

[46] De Vlieger D., Ballegeer M., Rossey I., Schepens B., Saelens X. (2019). Single-domain antibodies and their formatting to combat viral infections. Antibodies.

[47] Holliger P., Hudson P. (2005). Engineered antibody fragments and the rise of single domains. Nat. Biotechnol..

[48] Raj V., Okba N., Gutierrez-Alvarez J., Drabek D., van Dieren B., Widagdo W., Lamers M.M., Widjaja I., Fernandez-Delgado R., Sola I. (2018). Chimeric camel/human heavy-chain antibodies protect against MERS-CoV infection. Sci. Adv..

[49] Zhao G., He L., Sun S., Qiu H., Tai W., Chen J., Li J., Chen Y., Guo Y., Wang Y. (2018). A novel nanobody targeting middle east respiratory syndrome coronavirus (MERS-CoV) receptor-binding domain has potent cross-neutralizing activity and protective efficacy against MERS-CoV. J. Virol..

[50] Bobkov V., Zarca A., Van Hout A., Arimont M., Doijen J., Bialkowska M., Toffoli E., Klarenbeek A., van der Woning B., van der Vlient H.J. (2018). Nanobody-Fc constructs targeting chemokine receptor CXCR4 potently inhibit signaling and CXCR4-mediated HIV-entry and induce antibody effector functions. Biochem. Pharmacol..

[51] McEwan W., Tam J., Watkinson R., Bidgood S., Mallery D., James L. (2013). Intracellular antibody-bound pathogens stimulate immune signaling via the Fc receptor TRIM21. Nat. Immunol..

[52] Laursen N., Friesen R., Zhu X., Jongeneelen M., Blokland S., Vermond J., van Eijgen A., Tang C., van Diepen H., Obmolova G. (2018). Universal protection against influenza infection by a multidomain antibody to influenza hemagglutinin. Science.

[53] Hussack G., Ryan S., van Faassen H., Rossotti M., MacKenzie C., Tanha J. (2018). Neutralization of *Clostridium difficile* toxin B with VHH-Fc fusions targeting the delivery and CROPs domains. PLoS ONE.

[54] Sepulveda J., Mukherjee J., Tzipori S., Simpson L., Shoemaker C. (2010). Efficient serum clearance of botulinum neurotoxin achieved using a pool of small antitoxin binding agents. Infect. Immun..

[55] Miethe S., Rasetti-Escargueil C., Liu Y., Chahboun S., Pelat T., Avril A., Frenzel A., Schirrmann T., Thullier P., Sesardic D. (2014). Development of neutralizing scFv-Fc against botulinum neurotoxin A light chain from a macaque immune library. MAbs.

[56] Torgeman A., Ozeri E., Ben David A., Diamant E., Rosen O., Schwartz A., Barnea A., Makovitzki A., Mimran A., Zichel R. (2017). Role of homologous Fc fragment in the potency and efficacy of anti-botulinum antibody preparations. Toxins.

[57] Abbasova S.A.G., Ruddenko N.V., Gorokhovatskiĭ A.I., Kapralova M.V., Vinogradova I.D., Vertiev I.D.V., Nesmeianov V.A., Grishin E.V. (2011). Monoclonal antibodies to type A, B, E and F botulinum neurotoxins. Bioorg. Khimiia.

[58] Arbabi-Ghahroudi M., Desmyter A., Wyns L., Hamers R., Muyldermans S. (1997). Selection and identification of single domain antibody fragments from camel heavy-chain antibodies. FEBS Lett..

[59] Ledsgaard L., Kilstrup M., Karatt-Vellatt A., McCafferty J., Laustsen A. (2018). Basics of antibody phage display technology. Toxins.

[60] Hoogenboom H.R., Griffiths A.D., Johnson K.S., Chiswell D.J., Hudson P., Winter G. (1991). Multi-subunit proteins on the surface of filamentous phage: Methodologies for displaying antibody (Fab) heavy and light chains. Nucleic Acids Res..

